# Advances in research on RNA methylation and cancer radiotherapy resistance

**DOI:** 10.3389/fonc.2025.1596541

**Published:** 2025-07-31

**Authors:** Hui Liu, Hui Luo, Ming Jin, Zhen Zheng, Yang Xi, Kaitai Liu

**Affiliations:** ^1^ Department of Radiation Oncology, The Affiliated Lihuili Hospital of Ningbo University, Ningbo, Zhejiang, China; ^2^ Department of Biochemistry and Molecular Biology, School of Basic Medical Sciences, Health Science Center, Ningbo University, Ningbo, Zhejiang, China

**Keywords:** m6A methylation, m5C methylation, m7G methylation, m1A methylation, radioresistance

## Abstract

RNA methylation is a type of reversible chemical modification in epitranscriptomics that influences gene expression by dynamically regulating RNA functions. RNA methylation comprises N6-methyladenosine (m6A), 5-methylcytosine (m5C), N7-methylguanosine (m7G), N1-methyladenosine (m1A), and 3-methylcytosine (m3C) modifications. These are dynamically controlled by a tripartite enzymatic system: methyltransferases (“writers”) add methyl groups, demethylases (“erasers”) remove them, and RNA-binding proteins (“readers”) recognize and interpret the modifications to mediate downstream biological effects. Extensive research has shown the importance of RNA methylation in the onset and progression of cancer. RNA methylation contributes to radioresistance in cancer cells through various mechanisms, affecting therapeutic outcomes. To date, the precise functions of RNA methylation in cancer radioresistance remain unclear. This review summarizes recent advances in m6A, m5C, m7G, and m1A methylation in cancer radioresistance regulation and discusses the clinical potential of precision therapeutic strategies targeting these methylation modifications.

## 1 Introduction

Cancer represents a major challenge to public health, with an overall 19.74 million new diagnoses reported worldwide in 2022 alone. The International Agency for Research on Cancer (IARC) projects a 77% increase in annual global cancer cases by 2050, reaching approximately 35 million cases (1). Based on the updated cancer epidemiology statistics from the United States, the projected incidence of newly confirmed malignant neoplasms in 2025 is 2,041,910 cases, with an estimated 618,120 deaths attributable to malignancies (2).Current cancer treatment options include surgery, radiotherapy, chemotherapy, and novel targeted therapies (3). Among these, radiotherapy is crucial in cancer treatment, providing clinical benefits to over 50% of patients (4). Radiotherapy primarily involves inducing DNA double-strand breaks (DSBs) to damage cancer cells (5). However, radioresistance, characterized by cancer cells’ resistance to radiotherapy, reduces therapeutic efficacy and can lead to treatment failure (6), posing a significant challenge in managing malignant tumors. Notably, in pharmacotherapy, resistance to chemotherapy, targeted therapy, and immunotherapy has been established as closely associated with RNA modifications (7). Simultaneously, the mechanisms underlying radioresistance are complex, involving multiple biological processes. Research has identified several key factors contributing to the development of radioresistance in cancer cells, including improved DNA damage repair capacity, alterations in the tumor microenvironment (TME), activation of epithelial-mesenchymal transition (EMT), the presence of cancer stem cells (CSCs), regulation of autophagy, involvement of transcription factors such as nuclear factor-kappa B (NF-κB), signal transducer and activator of transcription 3 (STAT3), nuclear factor erythroid 2-related factor 2 (NRF2), and hypoxia-inducible factor 1 (HIF-1), as well as epitranscriptomics (8, 9). In recent years, RNA methylation, an important reversible chemical modification in the field of epitranscriptomics, has been closely associated with cancer cell radioresistance. RNA methylation affects cancer cell sensitivity to radiotherapy by regulating gene expression, RNA stability, and translation efficiency.

RNA methylation serves as a fundamental regulatory mechanism within the field of epitranscriptomics. It refers to the reversible chemical modifications of RNA nucleotides that dynamically regulate gene expression without altering the RNA sequence. This process encompasses several types of methylation, including N6-methyladenosine (m6A) methylation, 5-methylcytosine (m5C) methylation, N7-methylguanosine (m7G) methylation, N1-methyladenosine (m1A) methylation, and 3-methylcytosine (m3C) methylation. These modifications are found in various types of RNA, including mRNA, tRNA, and rRNA (10). RNA methylation regulates gene expression and has been linked to tumorigenesis and metastasis, as well as resistance to chemotherapy and radiotherapy. In cancer, RNA methylation is controlled primarily by methyltransferases, demethylases, and binding proteins. Methyltransferases transfer methyl groups to specific bases on the RNA, thus acting as “writers”. Demethylases are responsible for removing methyl groups, making RNA methylation dynamic and reversible, and thus serve as “erasers”. Binding proteins recognize and interact with methylated RNA, acting as “readers”, affecting RNA metabolism and function (10, 11). Together, these three components regulate RNA methylation status.

**Figure 1:**
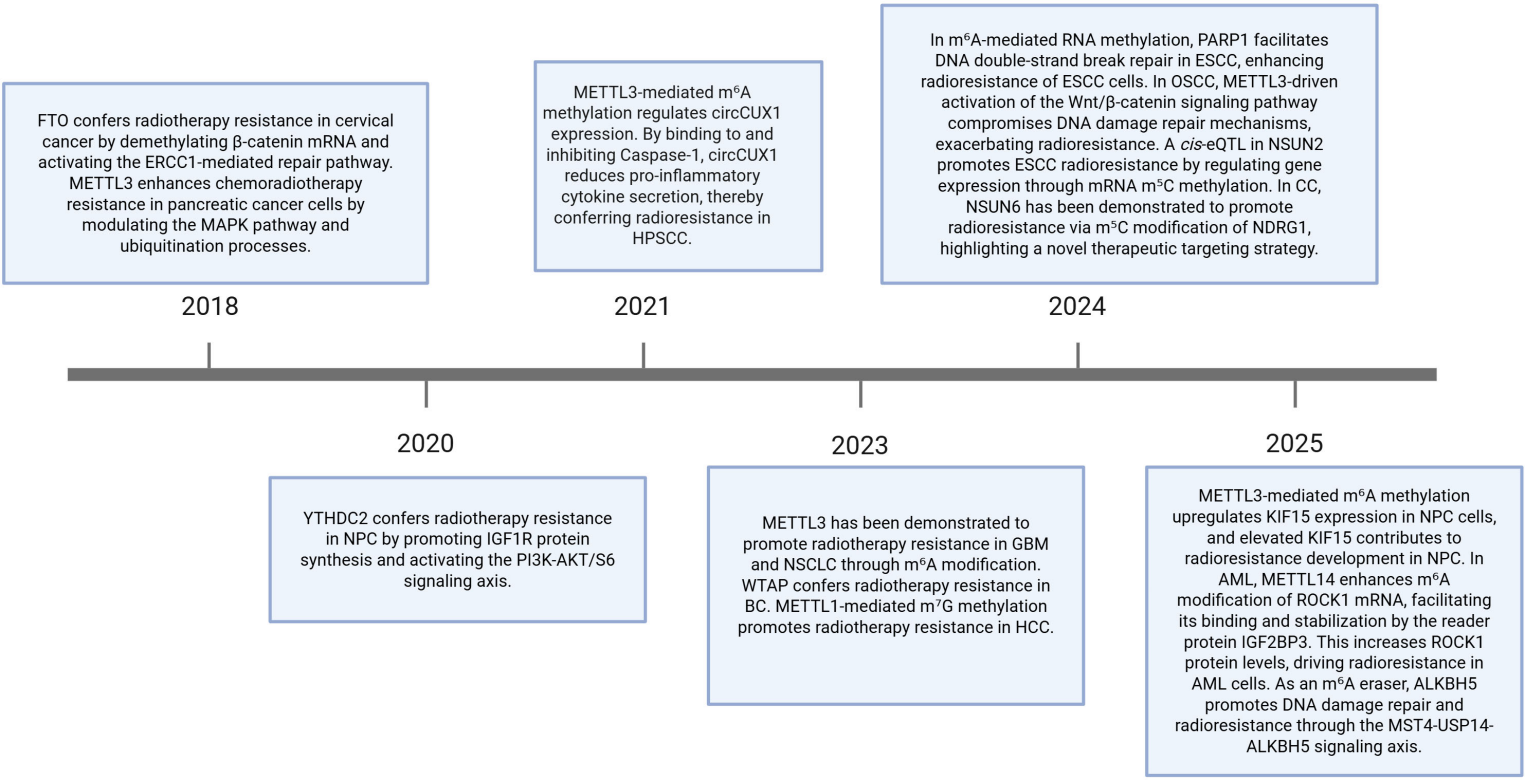
Recent advances in RNA methylation and cancer radioresistance.

This review summarizes recent advances in m6A, m5C, m7G, and m1A methylation in cancer radioresistance (**Figure 1**) and discusses the clinical potential of targeted therapeutic strategies aimed at these methylation modifications (**Table 1**).

**Table 1:** The regulatory role of RNA methylation (including m6A, m5C, m7G and m1A) in radioresistance across multiple cancer types.

Methylation	Function	Methylation module	Cancer	Target/Pathway	Mechanism	Cite
m6A methylation	writer	METTL3	GBM	Wnt/β-catenin Signaling Pathway	METTL3 promotes the self-renewal of glioma stem cells by regulating LINC00839 to activate the Wnt/β-catenin signaling pathway, leading to radiotherapy resistance in GBM.	(12)
			OSCC	SALL4	METTL3 activates the Wnt/β-catenin signaling pathway by regulating the SALL4 target, promoting the renewal of tumor stem cells, leading to radiotherapy resistance in OSCC.	(13)
			NSCLC	H2AX	METTL3 regulates the resistance of NSCLC to carbon ion radiotherapy by modulating H2AX expression and influencing signaling pathways such as PI3K/AKT and MAPK.	(14)
			PDAC	Genes associated with DNA damage repair; MAPK cascade, ubiquitin-dependent processes, and RNA splicing pathways.	METTL3 may confer radiotherapy resistance by influencing the expression of DNA damage repair genes, as well as through mechanisms such as the MAPK cascade, ubiquitin-dependent processes, and RNA splicing pathways.	(15)
			ESCC	LNCAROD	METTL3 confers radiotherapy resistance in ESCC cells by upregulating the expression of LNCAROD, inhibiting PARP1 degradation, and enhancing DNA double-strand break repair capacity.	(16)
			NPC	KIF15	METTL3-mediated m6A methylation can lead to increased expression of KIF15 in NPC cells, and the elevated expression of KIF15 is associated with radiotherapy resistance. Inhibition of KIF15 may alleviate radiotherapy resistance in NPC.	(17)
			HPSCC	circCUX1	METTL3 regulates the expression of the circCUX1 gene, and the suppression of circCUX1 expression reduces the release of inflammatory factors, leading to radiotherapy resistance.	(18)
		WTAP	BC	NRP1	WTAP downregulates the expression of Bcl-2 in BC by mediating m6A modification of NRP1, promoting stem cell-like properties and enhancing radiotherapy resistance in BC cells.	(19)
			GC	TGF-β	Overexpression of WTAP enhances radiotherapy resistance in GC cells by accelerating TGF-β-induced EMT.	(20)
		METTL14	ESCC	pri-miR-99a	METTL14 enhances the stem cell-like properties of cancer cells by promoting the maturation of pri-miR-99a and stabilizing miR-99a-5p, thereby conferring radioresistance to ESCC cells.	(21)
			EC	PRMT3	PRMT3-mediated METTL14 enhances cellular susceptibility to ferroptosis. Depletion of PRMT3 suppresses resistance to radiotherapy by promoting ferroptosis in EC cells.	(22)
	reader	YTHDC2	NPC	IGF1R	YTHDC2 promotes the translation of IGF1R, activating the PI3K-AKT/S6 signaling pathway, thereby conferring resistance to radiotherapy.	(23)
		YTHDF3	CC	HNF1-α	YTHDF3 promotes the translation of RAD51D to mediate HNF1-α regulation in CC radiotherapy resistance. Depletion of HNF1-α reduces, while its overexpression enhances, the resistance of CC cells to radiotherapy both *in vitro* and *in vivo*.	(24)
	eraser	FTO	NPC	OTUB1	FTO promotes the expression of the deubiquitinating enzyme OTUB1, a member of the OUT domain family, thereby suppressing radiation-induced ferroptosis and ultimately conferring resistance to radiotherapy in NPC.	(25) (26)
			CSCC	β-catenin	FTO regulates the expression of β-catenin, thereby enhancing resistance to chemoradiotherapy both *in vitro* and *in vivo*.	(27)
		ALKBH5	HCC	SOX4	ALKBH5 amplifies the characteristics of liver cancer stem cells by mediating the expression of SOX4 and activating the SHH signaling pathway, thereby conferring resistance to radiotherapy.	(28, 29)
m5C methylation	writer	TRDMT1	BC	TRDMT1–m5C-RAD52-RAD51	TRDMT1 interacts with FMRP to promote transcription-coupled homologous recombination through the TRDMT1–m5C-RAD52-RAD51 axis. Loss of FMRP and TRDMT1 increases the sensitivity of BC cells to radiation.	(30, 31)
		NSUN2	ESCC	STAT1	Increased activity of NSUN2 enhances the expression of STAT1 cis-eQTL, thereby promoting resistance to radiotherapy in ESCC cells.	(32)
		NSUN6	CC	NSUN6/ALYREF-m5C-NDRG1 pathway	Elevated expression of NSUN6 promotes resistance to radiotherapy in CC by activating the NSUN6/ALYREF-m5C-NDRG1 pathway.	(33)
	reader	FMRP	BC	TET1	FMRP interacts with TET1 to facilitate the DNA damage repair process, thereby promoting resistance to radiotherapy in BC cells.	(34) (31)
		ALYREF	CC	NSUN6/ALYREF-m5C-NDRG1 pathway	ALYREF binds to NDRG1 and participates in the NSUN6/ALYREF-m5C-NDRG1 pathway, promoting resistance to radiotherapy in CC.	(33)
m7G methylation	reader	METTL1	HCC	DNA-PKcs or DNA Ligase IV	METTL1 selectively regulates the translation of DNA-PKcs or DNA ligase IV, enhancing the efficiency of DSB repair and conferring resistance to ionizing radiation in HCC.	(35)
m1A methylation			LADC		m1A methylation may influence immune cell infiltration and function within the tumor microenvironment, thereby affecting sensitivity to radiotherapy.	(36)
			other	MYC/PD-L1 signaling pathway	m1A methylation downregulates the MYC/PD-L1 signaling pathway, thereby influencing the efficacy of radiotherapy.	(37)

## 2 m6A methylation and radiotherapy resistance

**Figure 2:**
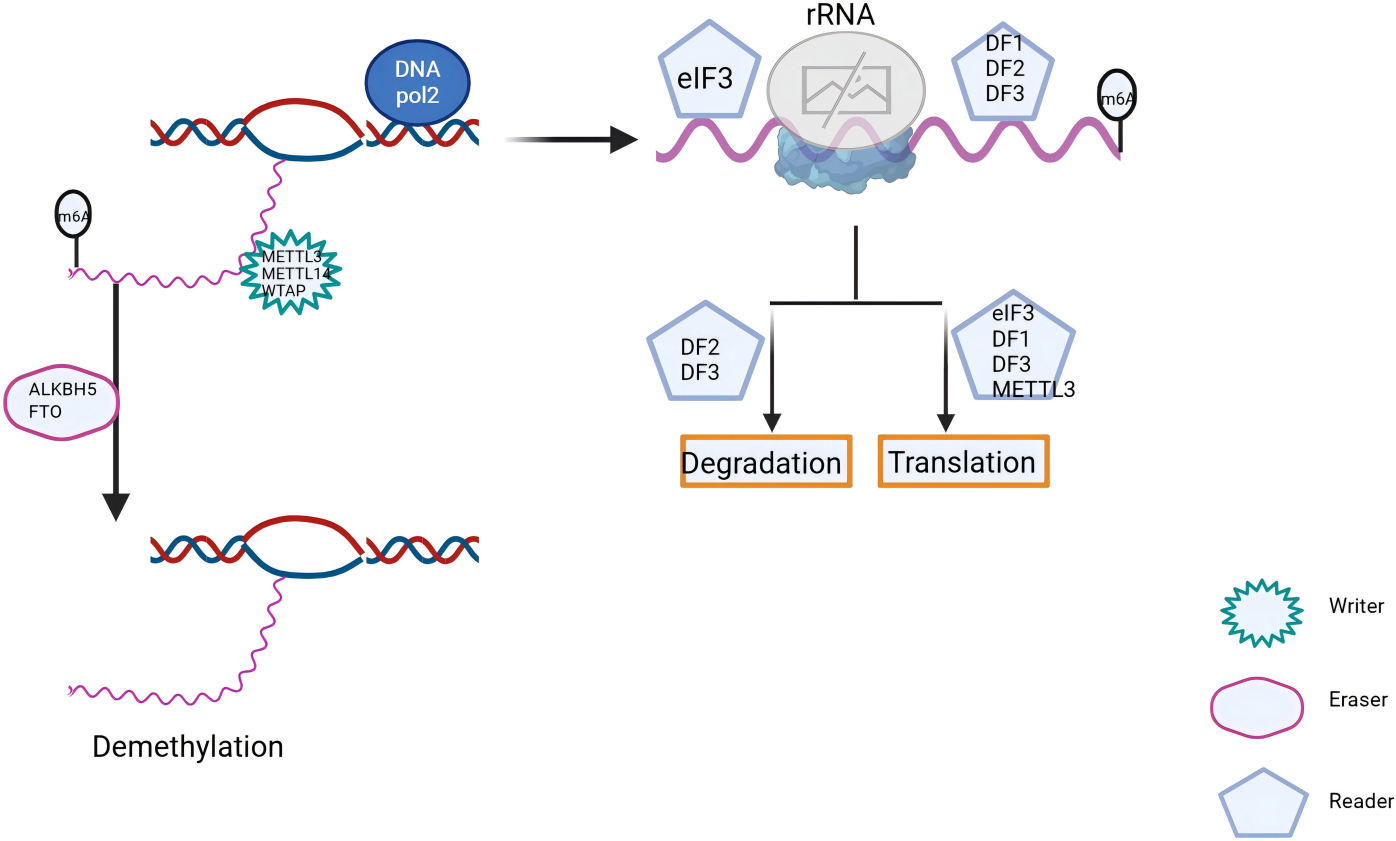
m6A RNA methylation is a dynamic and reversible epigenetic modification. Its regulation is primarily mediated by three key classes of proteins: “writers”, “erasers”, and” readers”. Through the coordinated actions of these proteins, m6A dynamically modulates mRNA and cellular functions, playing a pivotal role in cancer initiation, progression, and radiation resistance.

**Figure 3:**
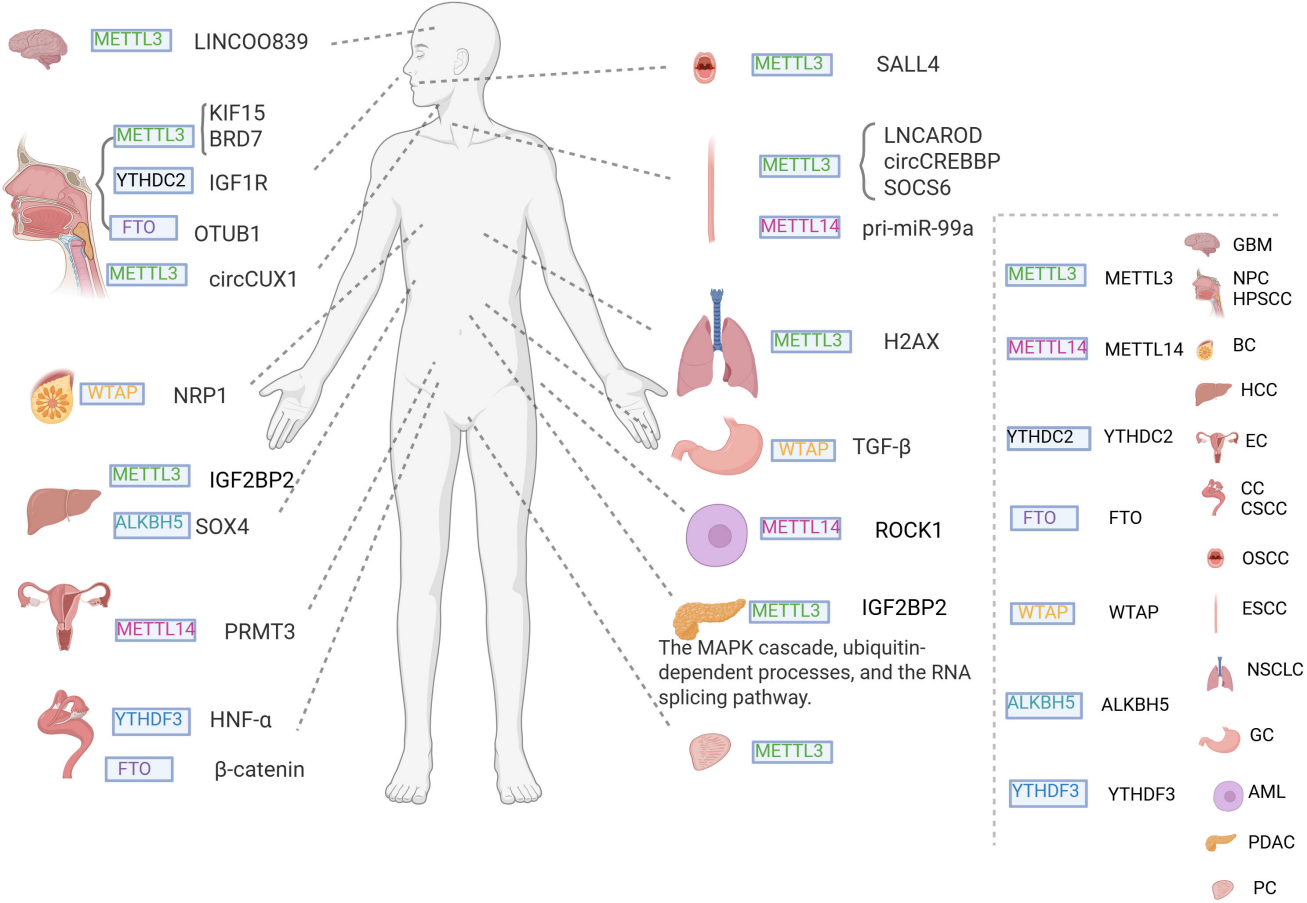
The role of m6A RNA modification in cancer radiotherapy resistance. m6A RNA modification is a dynamic and reversible process, and its associated proteins are implicated in the development of radiotherapy resistance in various cancers. Regulatory factors, known as “writers”, include METTL3, WTAP and METTL14; ’readers’ include YTHDC2 and YTHDF3; and ‘erasers’ include FTO and ALKBH5.

m6A methylation is a well-studied modification controlled by a tripartite system of “writers,” “erasers,” and “readers.” The “writers” are represented by methyltransferase-like 3 (METTL3) (38), METTL14 (39), METTL16 (40), Wilms tumor 1-associated protein (WTAP) (41), and VIRMA (42). The “erasers” include fat mass and obesity-associated protein (FTO) (43) and ALKB homolog 5 (ALKBH5) (44). The “readers” include YTHDC1 (45), YTHDC2 (46), YTHDF1 (47), YTHDF2 (48), and the heterogeneous nuclear ribonucleoprotein (HNRNP) family (49) (**Figure 2**). These m6A methylation regulators can promote or suppress cancer initiation and progression (50–52) and are also closely associated with cancer radioresistance (**Figure 3**).

### 2.1 METTL3

METTL3, one of the methyltransferases responsible for m6A methylation, has been shown to potentially contribute to cancer radioresistance by improving DNA repair capacity in malignant cells, promoting CSCs generation, and inducing tumor cell autophagy.

#### 2.1.1 Glioblastoma

METTL3 contributes to radioresistance in GBM by enhancing glioma stem cell (GSC) generation (12, 53). RNA immunoprecipitation (RIP) assays identified SOX2 as an authentic m^6^A substrate of METTL3. METTL3-mediated m^6^A modification stabilizes SOX2 mRNA, thereby maintaining the stemness properties of glioma stem cells (GSCs) and consequently driving radioresistance (53). Activation of the Wnt/β-catenin axis is closely associated with glioblastoma stem-like cells (GBM-SCs) formation (54). RNA-seq and m6A-seq analyses identified LINC00839, a long non-coding RNA (lncRNA), as a downstream target of METTL3. Further studies revealed that LINC00839 activates the Wnt/β-catenin signaling pathway by interacting with its key components, therefore supporting GSC self-renewal and promoting radioresistance in cancer cells (12).

#### 2.1.2 Oral squamous cell carcinoma

METTL3 regulates transcriptional targets, including SALL4. The transcriptional activation of SALL4 promotes β-catenin nuclear translocation and upregulates downstream target gene expression after radiotherapy, activating the Wnt/β-catenin signaling pathway (13). In OSCC, this pathway activation maintains CSC self-renewal and stemness and impairs DNA damage repair, exacerbating radioresistance in cancer cells (13).

#### 2.1.3 Lung cancer

METTL3-mediated m6A modifications of mRNA are closely linked to carbon ion radiotherapy resistance in Non-Small Cell Lung Cancer(NSCLC). METTL3 is upregulated in NSCLC, and its knockout enhances cellular sensitivity to radiotherapy. RNA-seq and m6A-seq analyses indicated that METTL3 regulates the expression of histone H2A family member X (H2AX) through m6A modification, affecting the PI3K/AKT and mitogen-activated protein kinase (MAPK) axes, which contribute to NSCLC resistance to carbon ion radiotherapy (14). Furthermore, recent investigations indicate that METTL3 overexpression promotes colony formation and proliferation in bystander cells of irradiated lung cancer, suppresses micronucleus formation kinetics, and attenuates DNA damage by regulating inflammatory responses (55). This suggests that reduced DNA damage may contribute to radiation resistance in LC.

#### 2.1.4 Pancreatic ductal adenocarcinoma

Studies have shown that METTL3 significantly increases radioresistance in PDAC cells by regulating m6A RNA modifications. Under low-dose irradiation, METTL3-knockdown cells exhibit increased radiosensitivity, suggesting that METTL3 may contribute to low-dose radiotherapy resistance. Mechanistically, METTL3 promotes radioresistance in PDAC by activating the MAPK cascade and processes involving ubiquitin and RNA splicing, which improves DNA damage repair. However, the precise mechanisms remain unclear and require further investigation (15). In PDAC, insulin-like growth factor 2 mRNA-binding protein 2 (IGF2BP2) upregulates the expression of polo-like kinase 1 (PLK1) by binding to m^6^A sites on PLK1 mRNA. Meanwhile, METTL3 maintains PLK1 expression through m^6^A methylation, thereby regulating the PDAC cell cycle. Disruption of the METTL3-IGF2BP2-PLK1 axis (for instance, through methylation inhibition) induces replication stress-induced cell death (56), presenting a novel therapeutic strategy to overcome radioresistance in PDAC.

#### 2.1.5 Esophageal squamous cell carcinoma

ESCC contributes to radioresistance by affecting DNA recombination repair and signaling pathway LNCAROD has been identified as a METTL3-mediated lncRNA. METTL3 significantly upregulates LNCAROD expression through m6A methylation. LNCAROD promotes the interaction between poly(ADP-ribose) polymerase 1 (PARP1) and nucleophosmin 1 (NPM1), preventing ubiquitin-proteasome-mediated degradation of PARP1. PARP1 is associated with DNA repair and facilitates the repair of DNA DSBs in ESCC, thus increasing ESCC radioresistance (16). Furthermore, METTL3 enhances radiosensitivity in ESCC through the m^6^A modification of circular CREBBP (circCREBBP). Mechanistically, METTL3-mediated m^6^A methylation of circCREBBP promotes its competitive binding with IGF2BP3, thereby inhibiting IGF2BP3-mediated stabilization of the oncogene MYC mRNA, which leads to the downregulation of MYC expression (57). Both *in vitro* and *in vivo* experiments confirm that the METTL3/circCREBBP/IGF2BP3/MYC axis reverses radioresistance. Genetic knockdown of circCREBBP significantly reduces MYC instability, enhances ESCC cell survival, and promotes radioresistance. Conversely, activation of this axis serves as a key therapeutic target for radiosensitization (57). Additionally, Ma et al. discovered that METTL3 catalytically elevates m^6^A modification levels in the 3’ UTR of SOCS6 mRNA, consequently inhibiting SOCS6 gene expression and blocking ferroptosis, which ultimately contributes to radioresistance in ESCC (58).

#### 2.1.6 Nasopharyngeal carcinoma

METTL3 serves as a master regulator of radioresistance in NPC, driving therapeutic resistance through three independent pathways. Its downstream targets—KIF15, the tumor suppressor BRD7, and SLC7A11—constitute a critical intervention network (17, 37, 59). Li et al. discovered that METTL3-mediated m^6^A methylation upregulates KIF15 expression in NPC cells, and this KIF15 overexpression contributes to radioresistance. Further studies demonstrate that inhibiting KIF15 expression alleviates NPC radioresistance by suppressing STAT3 activation and promoting autophagy (17). In parallel, the tumor suppressor BRD7 radiosensitizes NPC by disrupting USP5-METTL3 binding, which reduces METTL3 stability and inhibits BRCA1/RAD51-mediated DNA damage repair. Critically, clinical evidence confirms that high BRD7 and low METTL3 expression predict radiosensitivity and a favorable prognosis (37). Additionally, METTL3 stabilizes SLC7A11 mRNA via the m^6^A-IGF2BP2 axis, thereby driving NPC radioresistance through the suppression of ferroptosis. Mechanistically, METTL3-dependent SLC7A11 stabilization through this axis inhibits ferroptosis to confer radioresistance, whereas SLC7A11 knockdown or combined Erastin/radiotherapy reverses this effect (59).

#### 2.1.7 Hypopharyngeal squamous cell carcinoma

CircCUX1, a circular RNA derived from the *CUX1* gene, is upregulated in radiotherapy-resistant HPSCC where it is linked with shortened survival. m^6^A methylation by METTL3 was found to regulate circCUX1 expression, and circCUX1 knockdown increases the radiosensitivity of HPSCC cells. Moreover, circCUX1 interacts with Caspase1 to suppress its expression, preventing inflammatory factor production and contributing to radioresistance (18).

#### 2.1.8 Hepatocellular carcinoma

Studies demonstrate that the overexpression of METTL3 correlates with poor prognosis in HCC, whereas its knockdown significantly enhances radiosensitivity by inducing ferroptosis (60). Mechanistically, METTL3 mediates the m^6^A modification at the +1795 site of SLC7A11 mRNA, stabilizing transcripts through IGF2BP2 binding, while simultaneously inhibiting the ubiquitin-mediated degradation of SLC7A11 protein via the m^6^A/YTHDF2/SOCS2 axis.*In vivo* studies confirm that models with low METTL3/IGF2BP2 expression exhibit an enhanced response to radiotherapy. Importantly, the ablation of METTL3 abolishes the compensatory upregulation of SLC7A11 post-irradiation, which cooperatively promotes ferroptosis and radiosensitization. This work establishes the METTL3-IGF2BP2 axis as a potential therapeutic target for radiotherapy in HCC (60). These findings indicate that the inhibition of SLC7A11 ubiquitination through the m^6^A/YTHDF2/SOCS2 axis blocks radiation-induced ferroptosis, ultimately leading to radioresistance.

### 2.2 WTAP

The methyltransferase WTAP enhances stemness and EMT in tumor cells, which in turn increases radioresistance. NRP1, a transmembrane glycoprotein, is highly expressed across multiple cancer types. Studies have shown that radiotherapy alone significantly increases double-strand DNA (dsDNA) damage in breast cancer (BC) cells, whereas NRP1 overexpression combined with radiotherapy does not significantly affect dsDNA breaks, indicating that NRP1 plays a key role in BC radioresistance. Mechanistically, NRP1 downregulates Bcl-2 expression in BC through WTAP-mediated m6A modification, thus reducing radiation-induced apoptosis, promoting stemness in BC cells, and increasing their radioresistance (19). Similarly, Liu et al. reported that, after irradiation, WTAP overexpression in gastric cancer (GC) cells promotes EMT by accelerating TGF-β signaling, increasing radioresistance, whereas WTAP downregulation reduces radioresistance (20). Additionally, WTAP stabilizes SQLE mRNA expression through an m^6^A-dependent mechanism, enhancing CSC properties in high-grade serous ovarian carcinoma (HGSOC), which may indirectly contribute to radioresistance (61). These studies demonstrate that WTAP regulates downstream gene expression through m6A modification across multiple cancer types, affecting radioresistance.

### 2.3 METTL14

METTL14 is a well-characterized m6A regulator. In ESCC cells, METTL14 promotes pri-miR-99a maturation and miR-99a-5p stability, enhancing stemness in cancer cells and increasing radioresistance (21). Additionally, METTL14 mediates the regulation of radioresistance through ferroptosis pathways. Studies demonstrate that METTL14 reverses radioresistance in ESCC by promoting ferroptosis via enhanced m^6^A modification of ACSL4 (62). In endometrial cancer (EC), protein arginine methyltransferase 3 (PRMT3)-mediated METTL14 promotes ferroptosis sensitivity by reducing the expression and stability of glutathione peroxidase 4 (GPX4). Further studies have revealed that PRMT3 inhibition increases radiosensitivity, whereas PRMT3 depletion suppresses radioresistance by promoting ferroptosis in EC (22). In acute myeloid leukemia (AML), AML-derived mesenchymal stem cells (AML-MSCs) deliver METTL14 to leukemia cells via exosomes, where it stabilizes ROCK1 expression through the m^6^A-IGF2BP3 axis, thereby mediating radioresistance. Specifically, exosome-transferred METTL14 enhances the m^6^A modification of ROCK1 mRNA, facilitating its binding to and stabilization by the reader protein IGF2BP3. Consequently, ROCK1 protein levels are upregulated, driving AML cell proliferation and contributing to radioresistance (63).

### 2.4 YTHDC2

Research indicates that the m^6^A reader protein YTHDC2 plays a critical role in radioresistance across various malignancies, with IGF1R acting as the central hub mediating YTHDC2-driven therapeutic resistance. In NPC, previous studies demonstrate that IGF1R inhibition, such as through Linsitinib, blocks downstream Akt/ERK phosphorylation, suppresses proliferation, induces apoptosis, and significantly radiosensitizes tumors by reversing resistance. This highlights the therapeutic targeting potential of IGF1R and establishes a definitive association between IGF1R and NPC radioresistance (64). Further investigations reveal that YTHDC2 is highly expressed in radioresistant NPC cells and clinical specimens. It facilitates the translation of IGF1R, activating the PI3K-AKT/S6 signaling pathway to confer radioresistance, thereby emerging as a promising therapeutic target for NPC radiosensitization. Experimentally, the depletion of YTHDC2 downregulates IGF1R expression and suppresses PI3K-AKT/S6 signaling, consequently alleviating radioresistance in NPC cells (23). In neuroblastoma (NB), the activation of the STAT3/AKT axis stimulates CSC properties and EMT, both of which are intrinsically linked to radioresistance. Although the response to radiotherapy remains untested in NB, the identified IGF1R-kinase signaling (STAT3/AKT)-CSC/EMT mechanistic logic aligns with the NPC axis, demonstrating a shared dependence on IGF1R-driven downstream pathways to sustain resistance phenotypes (65). Therefore, targeting YTHDC2, IGF1R, or their downstream kinases (PI3K/AKT, STAT3) represents a viable strategy to overcome radioresistance and achieve radiosensitization by suppressing CSC traits and aberrant signaling cascades.

### 2.5 YTH domain-containing family protein 3

The m6A “reader” protein YTHDF3 contributes to radiotherapy resistance by modulating gene expression. A study by Du et al. found that the levels hepatocyte nuclear factor 1-alpha (HNF1-α) are markedly higher in radiotherapy-resistant cervical cancer (CC) tissues and cell lines. This upregulation increases the transcription of YTHDF3, leading to m6A modifications of RAD51D mRNA. Furthermore, YTHDF3 mediates HNF1-α-regulated radiotherapy resistance in CC by promoting m6A-dependent translation of RAD51D translationr. Depletion of HNF1-α reduces radiotherapy resistance, whereas its overexpression enhances it in CC cells and tissues. In summary, YTHDF3 affects radiotherapy resistance in CC cells (24).

### 2.6 FTO

FTO, a critical RNA m^6^A demethylase, plays a pivotal role in radioresistance across diverse malignancies. It drives therapeutic resistance through epitranscriptomic regulation of downstream effectors, including CSC properties, EMT, DNA repair, and oncogenic signaling pathways.Studies demonstrate that in colorectal cancer, cytoplasmic FTO suppresses CSC phenotypes via its m^6^A demethylase activity. Conversely, low FTO expression induces m^6^A hypermethylation, significantly enhancing the *in vivo* tumorigenicity and radioresistance of CSCs (66). In lung adenocarcinoma (LUAD), FTO stabilizes PHF1 mRNA through demethylation, forming a tumor-suppressive axis. The downregulation of the FTO/PHF1 axis promotes tumor cell self-renewal, progression, and poor prognosis by enhancing FOXM1 expression, thereby compromising therapeutic efficacy (67). Breast cancer research reveals that chemotherapy-induced senescent neutrophils upregulate intratumoral FTO via exosomal piR-17560 secretion. Elevated FTO subsequently reduces m^6^A modification on ZEB1 mRNA, stabilizing its transcript and promoting EMT and radioresistance (68). In glioblastoma (GBM), pharmacological inhibition of FTO (e.g., using FB23-2) increases m^6^A modification on the target gene VEGFA, downregulating its expression and impairing DNA damage repair (e.g., sustaining γH_2_AX foci and reducing Rad51 recruitment). This significantly enhances the radiosensitivity of glioblastoma stem cells (GSCs), suppresses tumor growth, and prolongs survival, indicating that FTO upregulation promotes GBM radioresistance (69). Additionally, FTO in NPC promotes OTUB1 expression by erasing m^6^A marks on OTUB1 transcripts, suppressing radiation-induced ferroptosis (25), and induces CD44 splice variant switching (CD44v) via lncRNA-HOTAIRM1 interaction to inhibit ferroptosis, collectively driving radioresistance (26). In CSCC, FTO upregulates β-catenin expression by reducing the m^6^A levels of its mRNA, thereby enhancing chemoradioresistance both *in vitro* and *in vivo* (27). Collectively, FTO acts as a core determinant of pan-cancer radioresistance, positioning it as a promising therapeutic target for overcoming resistance and improving the efficacy of radiotherapy.

### 2.7 ALKBH5

ALKBH5, an m6A “eraser”, increases radiotherapy resistance in hepatocellular carcinoma (HCC) and GBM by influencing CSCs. CSCs are closely linked to cancer therapy resistance through various pathways, including activating DNA damage repair processes, the EMT, and modulating the levels of genes associated with self-renewal (28, 29). Studies demonstrate that elevated expression of ALKBH5 enhances glioblastoma (GBM) radioresistance by modulating homologous recombination (HR) (70). Further investigations confirm that glioblastoma stem cells (GSCs) are the primary source of radioresistance in GBM, with the MST4-USP14-ALKBH5 signaling axis serving as its core mechanism. Specifically, ALKBH5 undergoes deubiquitination mediated by USP14 (a deubiquitinase), which confers protein stability that is further potentiated by phosphorylation from the upstream kinase MST4. This pathway sustains GSC stemness and tumorigenicity, while robustly promoting DNA damage repair and driving therapeutic radioresistance (71). Liver cancer stem cells (LCSCs) exhibit CSC-like properties and significantly affect HCC progression and therapeutic resistance (72). ALKBH5 is upregulated in LCSCs, where it promotes SOX4 expression through demethylation. The ALKBH5/SOX4 axis enhances LCSC properties by the activation of SHH signaling (73). In summary, ALKBH5 overexpression in HCC may contribute to radiotherapy resistance.

### 2.8 Others

In NSCLC, m6A RNA methylation-mediated regulation of mitochondrial RNA-processing endoribonuclease (RNase MRP) enhances the properties of cancer stem cells and promotes the EMT through the TGFB/SMAD2/SMAD3 pathway, thus contributing to radiotherapy resistance (74). Meanwhile, m6A-modified enhancer RNAs (eRNAs) are closely linked to the progression of bone-metastatic prostate cancer (mPCa) and its resistance to radiotherapy. Zhao et al (75) employed RNA sequencing and other methods to identify the m6A-modified bone-specific eRNA, MLXIP, associated with radiotherapy resistance. This eRNA inhibits RNA degradation by facilitating the interaction between the RNA-binding protein KHSRP and mRNA, affecting PC progression and its sensitivity to radiotherapy.

## 3 m5C methylation and radiotherapy resistance

**Figure 4:**
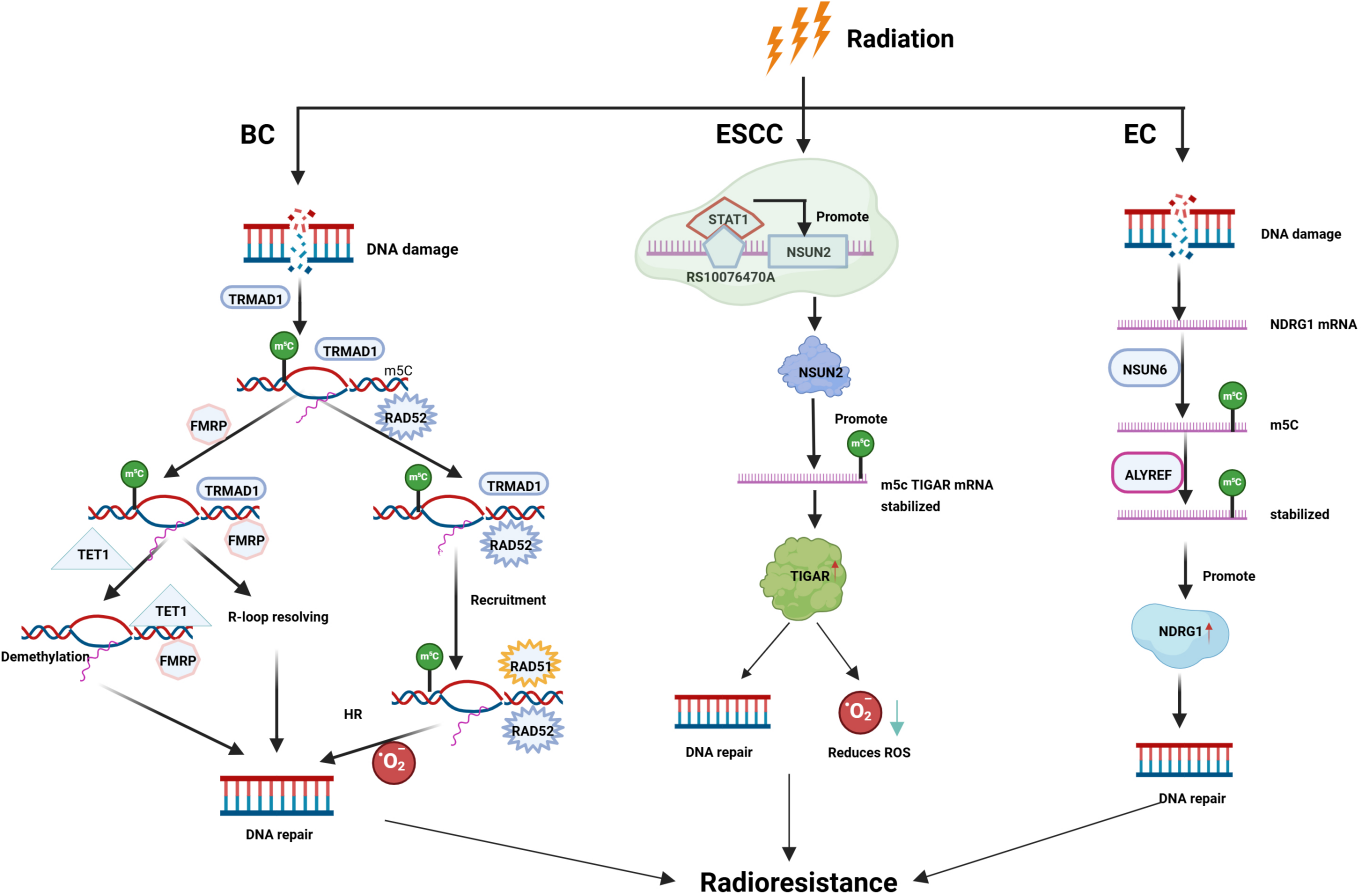
The role of m5C RNA methylation in tumor resistance to radiotherapy. m5C modification contributes to radioresistance in EC, BC, and ESCC by regulating mRNA stability, DNA damage repair, and epigenetic modulation.

m5C modification refers to the methylation of the fifth cytosine carbon in RNA and is commonly observed in RNA types such as mRNA, tRNA, rRNA, and enhancer RNA (11). The m5C methyltransferases are associated with both the TRDMT (76) and NSUN families (34, 77). Known “erasers” of m5C methylation include the TET enzyme family (30), while “readers” include fragile X messenger ribonucleoprotein (FMRP) (30), ALYREF (78), and YBX1 (79). m5C methylation regulators affect RNA stability, translation efficiency, and other processes, regulating various biological functions, including proliferation, differentiation, and apoptosis. They also play a key role in radiotherapy resistance in malignant tumors (80), primarily by improving DNA repair capacity and regulating gene expression, ultimately reducing cell death and leading to radiotherapy resistance (81, 82) (**Figure 4**).

### 3.1 FMRP

FMRP is an m5C “reader” that recognizes and binds to m5C-modified RNA. Through its interaction with the m5C eraser ten-eleven translocation protein 1 (TET1), FMRP induces the demethylation of m5C RNA modifications, therefore promoting mRNA-dependent DNA damage repair processes (34). Furthermore, FMRP interacts with the m5C methyltransferase TRDMT1, which facilitates transcription-coupled homologous recombination at reactive oxygen species (ROS)-induced DSBs through the TRDMT1– m5C-RAD52-RAD51 axis (31). The absence of FMRP and TRDMT1 increases radiation sensitivity in BC cells (30, 31), and BC cells with low TRDMT1 expression exhibit greater sensitivity to radiotherapy (31).

### 3.2 NSUN6

The mechanistic and functional diversity of NSUN6-mediated tumor radioresistance operates through the m^5^C-NDRG1 axis. Yu et al. discovered that in cervical cancer, NDRG1, as a transcriptional regulatory target of NSUN6, participates in radioresistance mechanisms. Elevated NSUN6 expression initiates the NSUN6/ALYREF-m^5^C signaling cascade, enhancing NDRG1 stability by augmenting its m^5^C RNA methylation levels, ultimately conferring radioresistance (33). Given that NDRG1 significantly promotes tumor progression and brain metastasis in aggressive breast cancer (83), NSUN6 may potentially co-drive breast cancer radioresistance via NDRG1 regulation. However, the molecular interactome of this signaling axis in breast cancer remains to be elucidated further. Conversely, NDRG1 exhibits context-dependent functional reversal in HCC, where it significantly suppresses HCC tumorigenesis and metastasis by inducing tumor cell ferroptosis (84). This highlights the cancer type-dependent biological effects mediated by NDRG1.Whether the NSUN6-m^5^C-NDRG1 axis universally drives pan-cancer radioresistance requires.

### 3.3 NSUN2

NSUN2, an m5C “writer,” participates in radiotherapy resistance by regulating gene expression. Niu et al. found that cis-expression quantitative trait loci (cis-eQTLs) in NSUN2 promote radiotherapy resistance in ESCC through mRNA-m5C methylation. Mechanistically, the *NSUN2* rs10076470 G-to-A mutation acts as a cis-eQTL for STAT1, a key transcription factor that is markedly upregulated in ESCC. This genetic variation increases NSUN2 activity, leading to enhanced m5C methylation and upregulation of multiple cancer-related genes, promoting ESCC progression and increasing resistance to radiotherapy (32).

## 4 m7G methylation and radiotherapy resistance

The m7G modification is frequently seen in tRNA, rRNA, and mRNA across both eukaryotic and prokaryotic organisms. m7G methylation primarily occurs at position 46 of tRNA and within the mRNA 5′ cap structure. The key regulators of m7G methylation include “writers” such as Trm8/Trm82 (85) and METTL1/WDR4 (86). FTO, primarily known as an m6A demethylase, also functions as an “eraser” of m7G methylation, affecting RNA stability and translation efficiency (87). Known “readers” include the QKI family (88) and YTH domain-containing proteins, which recognize m7G modifications and regulate RNA stability and translation (89).

**Figure 5:**
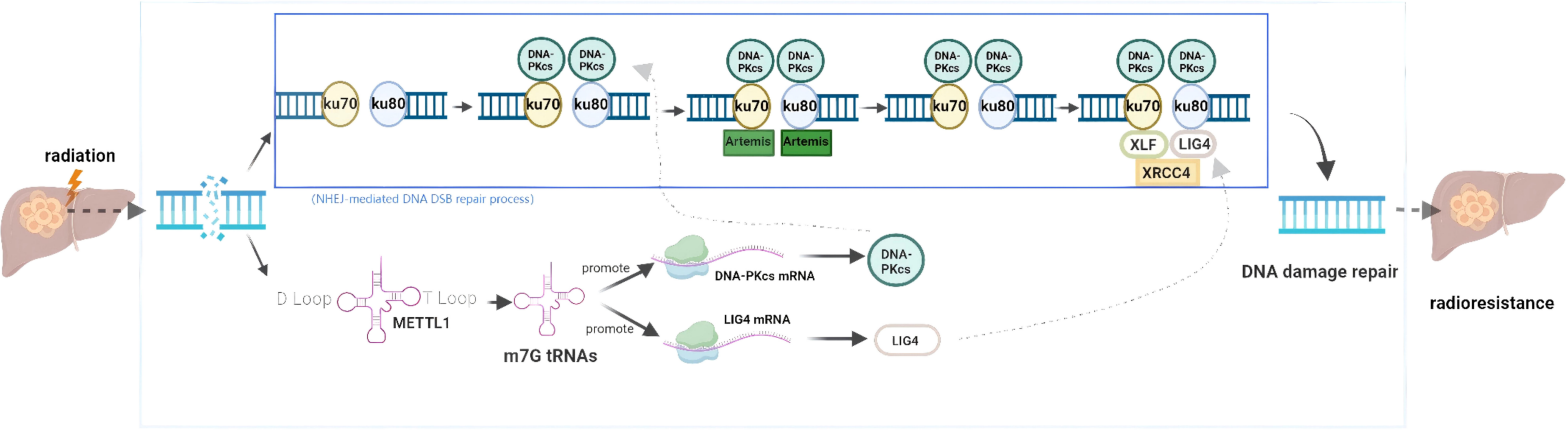
The role of m7G RNA modification in radiotherapy resistance of hepatocellular carcinoma. The key factor METTL1 promotes DNA damage repair in hepatocellular carcinoma cells through non-homologous end joining (NHEJ).

METTL1, involved in m7G tRNA modification, serves as a “writer” of m7G methylation. It is closely associated with tumorigenesis, progression, and resistance to radiotherapy. For instance, studies have indicated that increased METTL1 expression elevates the risk of neuroblastoma tumorigenesis (90) and promotes the growth and metastasis of NPC both *in vitro* and *in vivo* (91). Furthermore, Studies have shown that METTL1 is upregulated in various cancers, with its levels correlating with cancer malignancy. In HCC, ionizing radiation induces METTL1-mediated m7G tRNA methylation, selectively increasing the translation of DNA-dependent protein kinase catalytic subunit (DNA-PKcs) or DNA ligase IV through higher-frequency m7G-associated codons. This regulation enhances the DNA DSB repair through nonhomologous end joining (NHEJ), thus conferring resistance to ionizing radiation in HCC (35) (**Figure 5**).

## 5 m1A methylation and radiotherapy resistance

The m1A modification involves adenosine methylation the 1-position, affecting RNA structure and function. In eukaryotes, the methyltransferases (“writers”) responsible for m1A methylation primarily include TRMT10C, TRMT61B, TRMT61A, TRMT6, SDR5C1, and NML. The demethylases (“erasers”) mainly consist of α-ketoglutarate-dependent dioxygenases such as ALKBH7, ALKBH3, ALKBH1, and FTO. The known “readers” of m1A-modified RNA include YTHDF1-3 and YTHDC1 (11). Currently, direct causal evidence for the regulatory factors driving tumor radioresistance remains insufficient. Nevertheless, cutting-edge research has suggested their potential roles. For instance, the RNA demethylase ALKBH3 has been reported to influence radiation sensitivity by modulating the TME (92). These preliminary findings underscore the necessity for in-depth mechanistic dissection of relevant regulatory pathways in radioresistance.

m1A methylation contributes to radiotherapy resistance by modulating the TME, regulating gene expression, and altering cellular metabolic processes. Xu et al. reported that m1A methylation may affect the sensitivity of lung adenocarcinoma (LADC) cells to radiotherapy by affecting immune cell infiltration and function within the TME (36). Specifically, inhibition of m1A downregulates the MYC/PD-L1 axis involved in immune evasion of tumors. Since radiotherapy resistance has close associations with changes in the tumor immune microenvironment, m1A methylation may affect radiotherapy efficacy by modulating this signaling pathway (93).

## 6 Clinical significance of RNA methylation in radiotherapy resistance

RNA methylation holds significant clinical implications for radiotherapy resistance in cancer. Its levels can serve as biomarkers for predicting radiotherapy efficacy, helping in the identification of radiotherapy-resistant patients, and guiding personalized treatment strategies (94). Furthermore, RNA methylation-related enzymes, such as METTL3 and YTHDC2, may serve as therapeutic targets to overcome radiotherapy resistance. Modulating RNA methylation levels through inhibitors holds the potential for improving radiotherapy outcomes (13, 23). RNA methylation modifications influence radiotherapy efficacy by influencing DNA repair, tumor cell radiation sensitivity, and the tumor immune microenvironment. Therefore, precision treatment strategies based on RNA methylation research offer the potential to mitigate radiotherapy resistance and improve patient outcomes, representing a promising therapeutic approach (27).

### 6.1 Biomarkers

RNA methylation can serve as a biomarker for identifying malignant tumors resistant to radiotherapy. In GBM, METTL3 expression is associated with radioresistance, and its downregulation reduces DNA damage repair and increases radiosensitivity (12). Similarly, in NSCLC, METTL3 is upregulated, and its knockout increases cellular sensitivity to radiotherapy (14). These findings suggest that RNA methylation levels can function as biomarkers to predict radiotherapy efficacy, facilitating the identification of radiotherapy-resistant patients and enabling individualized precision treatment.

### 6.2 Therapeutic targets

Enzymes involved in RNA methylation modifications and their downstream regulatory targets hold significant therapeutic potential for overcoming radiotherapy resistance across various cancers. Studies have shown that METTL3 increases radiotherapy resistance in OSCC by targeting SALL4 (13), while METTL3 knockdown increases PDAC cell sensitivity to low-dose radiotherapy, suggesting its possible application as a target in treating the disease (15). In NPC, METTL3-mediated m6A methylation upregulates KIF15 expression, contributing to radiotherapy resistance. Inhibiting KIF15 expression has been found to mitigate this resistance (17). Collectively, these findings suggest that METTL3 is a promising therapeutic target for radiosensitization across various cancer types. Currently, small-molecule inhibitors targeting METTL3 are undergoing preclinical investigation (95), which may inform future combinatorial radiosensitization strategies. Similarly, in CSCC, FTO promotes β-catenin expression by reducing m6A modification levels, aggravating radiotherapy resistance. This suggests that targeting FTO or β-catenin may optimize therapeutic outcomes (27). However, FTO exhibits broad substrate specificity. Targeting FTO may influence the expression of metabolism-related genes, potentially resulting in metabolic dysregulation. This underscores the necessity for more precise strategies to target RNA methylation (69, 96). Furthermore, m5C modification-related proteins, including FMRP and members of the NSUN family, as well as the key m7G tRNA modification enzyme METTL1, have been reported to be involved in regulating the radiotherapy response in malignant tumors. Inhibiting the activity of these modification enzymes has been shown to improve radiotherapy efficacy in killing cancer cells (30–33). These findings not only highlight the key role of RNA methylation modifications in radiotherapy resistance but also provide diverse potential targets for developing precision radiotherapy sensitization strategies based on RNA modification regulation, offering significant clinical implications.

### 6.3 Combination

Combining methylation-modulating inhibitors with radiotherapy has been shown to suppress tumor growth and progression. Research indicates that STM2457, a novel inhibitor targeting METTL3, exhibits significant efficacy in preclinical models of AML (97). To evaluate its anti-leukemic effects in conjunction with radiotherapy, experiments were conducted using METTL3-knockout cells and murine models. The results demonstrate that the targeted inhibition of METTL3 by STM2457, when combined with *in vivo* radiotherapy, synergistically suppresses tumor growth (95). Furthermore, Zhang et al. found that inhibiting METTL3 enhances the radiosensitivity of HCC by activating the radiation-induced ferroptosis pathway (60). Additionally, studies have shown that the FTO inhibitor FB23-2, when combined with radiotherapy, significantly inhibits tumor spheroid formation and the self-renewal capacity of GSCs, suppresses cell proliferation, and induces apoptosis in GBM cells. Animal experiments further confirmed that FB23-2 combined with radiotherapy effectively inhibits intracranial tumor growth in mice (69). Collectively, these findings suggest that the targeted inhibition of METTL3 and FTO, in combination with radiotherapy, enhances the suppression of tumor growth and progression.

Moreover, studies have indicated that cyclooxygenase-2 (COX-2) is a potential target for radioprotection and radiosensitization. Inhibition of COX-2 (e.g., celecoxib) can reduce the resistance of malignant tumor cells to radiotherapy (98). In NPC, the resistance to radiotherapy is primarily mediated by EBV-encoded products (such as LMP1) and non-coding RNAs (miRNA/lncRNA/circRNA), which inhibit DNA damage repair, activate anti-apoptotic pathways (such as PI3K/AKT, NF-κB), and promote EMT. Combined chemoradiotherapy or targeting EBV/non-coding RNAs (e.g., olaparib inhibiting miR-519d, curcumin downregulating lncRNA AK294004) can reverse radiotherapy resistance (99).

In summary, targeting epigenetic regulation (such as METTL3, FTO, COX-2 inhibitors) or viral/non-coding RNA pathways (such as EBV-LMP1, miRNA/lncRNA), in conjunction with radiotherapy, can significantly enhance antitumor efficacy through synergistic mechanisms, providing new strategies to reverse radiotherapy resistance.

## 7 Conclusion

In summary, RNA methylation plays a crucial role in tumor radioresistance by regulating DNA damage repair and key signaling pathways. Current research has preliminarily elucidated the mechanisms of m6A; however, several limitations remain: the associations of other modifications such as m5C, m7G, and m1A with radioresistance have yet to be clarified. Additionally, the synergistic effects, targeting, and toxicity issues of methylation inhibitors (e.g., FTO/METTL3 targeted drugs) urgently need breakthroughs, and there is a lack of clinical validation. Furthermore, existing RNA methylation detection technologies exhibit insufficient sensitivity, limiting their clinical application as biomarkers. To address these limitations, future research should deeply explore the mechanisms of non-m6A modifications, advance human trials and safety optimization of inhibitors, and develop high-sensitivity multidimensional methylation detection systems, ultimately achieving precise design of individualized radiotherapy sensitization strategies.
